# The integration of weighted human gene association networks based on link prediction

**DOI:** 10.1186/s12918-017-0398-0

**Published:** 2017-01-31

**Authors:** Jian Yang, Tinghong Yang, Duzhi Wu, Limei Lin, Fan Yang, Jing Zhao

**Affiliations:** 1 Department of Mathematics, Logistical Engineering University, Chongqing, China; 2 Institute of Interdisciplinary Complex Research, Shanghai University of Traditional Chinese Medicine, Shanghai, China

**Keywords:** Gene association network, Weighted network, Link prediction, Network integration

## Abstract

**Background:**

Physical and functional interplays between genes or proteins have important biological meaning for cellular functions. Some efforts have been made to construct weighted gene association meta-networks by integrating multiple biological resources, where the weight indicates the confidence of the interaction. However, it is found that these existing human gene association networks share only quite limited overlapped interactions, suggesting their incompleteness and noise.

**Results:**

Here we proposed a workflow to construct a weighted human gene association network using information of six existing networks, including two weighted specific PPI networks and four gene association meta-networks. We applied link prediction algorithm to predict possible missing links of the networks, cross-validation approach to refine each network and finally integrated the refined networks to get the final integrated network.

**Conclusions:**

The common information among the refined networks increases notably, suggesting their higher reliability. Our final integrated network owns much more links than most of the original networks, meanwhile its links still keep high functional relevance. Being used as background network in a case study of disease gene prediction, the final integrated network presents good performance, implying its reliability and application significance. Our workflow could be insightful for integrating and refining existing gene association data.

**Electronic supplementary material:**

The online version of this article (doi:10.1186/s12918-017-0398-0) contains supplementary material, which is available to authorized users.

## Background

In cells, genes and their products usually perform particular cellular task and carry out their biological functions by interacting or communicating with each other [1]. Such interactions can be expressed with molecular networks [2] with different meaning at different levels. Specifically, at genomic level, gene regulatory networks are collections of interactions between transcription factors and their target genes in the process of regulating the gene expression levels of mRNA and proteins [3, 4]; while the co-expression relationships between genes can be described as gene co-expression networks [5]. At proteomic level, protein-protein interaction (PPI) networks [6, 7] represent the physical interactions between proteins. Generally, all of such functional interplays between genes can be integrated to construct a gene association network [8].

These years, high-throughput biological experiments have produced huge number of data concerning interactions between genes and their products, such as gene regulatory, gene co-expression, protein complex, and PPI data, based on which we can build gene association networks. However, there are two problems in the current experimental data. First, the known data is far from complete [9]. For example, it is estimated that experimentally confirmed human protein-protein interactions account for only 0.3% of the actual existence [10]. Second, high-throughput experiments usually produce large amount of false-positive and false-negative data [11].

To overcome the problem of data insufficiency, some research combined several databases to construct a larger network. For example, PPI data in the Entrez Gene database of NCBI is a combination of PPI data from different resources, such as HPRD [12], BioGrid [13] and BIND [14]. A human signaling network was constructed through combining human pathway data sources such as BioCarta [15], CST Signaling pathways [16], Pathway Interaction database (PID) [17], iHOP [18], and manual curation of human signaling network data from literature [19, 20]. This method can partly solve the problem of data scarcity. On the other hand, some studies applied link prediction [21] approach to de-noise the PPI networks [22, 23]. For each pair of nodes, this class of methods first utilized topology of the original PPI network to calculate a score which quantifies the existence likelihood of a link between the two nodes. Then they ranked all pairs in descending order of their scores, took out the same number of pairs with the highest ranks as in the original network, and linked these node pairs to reconstruct a new network. This new network was considered as a de-noised PPI network of the original one. However, such methods discarded quite large part of original links and added many new links. It is unavoidable that some discarded could be real links while some new are false links.

Another class of works uses computational approach, such as log likelihood ratio and naïve Bayesian network, to integrate heterogeneous biological evidence which is possible to reflect associations between genes [24–29]. The functional associations between genes are predicted and their confidence scores are obtained according to biological features of the gene pairs and the relationship with gold-standard positive and negative datasets. In this way, a meta-database that maps all interaction evidence onto a common set of genes is set up. Then we can construct a weighted gene association meta-network from such a database, where the link weight is the confidence score. This method, to some extent, can solve both of the problems mentioned above. However, in this framework of integrating multiple data resources, different research chose different data resources, gold-standard datasets and conducted prediction from scratch, not using results of other research. Although we can see some overlaps of data resources used in different study, their results show great difference. For example, our earlier study found that three existing weighted human gene association networks constructed in such way have almost same node sets, but they contain only a very small amount of common links [30].

In this work, based on existing networks, we propose a workflow to construct a weighted human gene association network that includes more links and more precise information. We focus our study on two weighted specific PPI networks (hsaPPI and Corum) and four gene association meta-networks (HumanNet, String, FunCoup and FLN). First, for each of the six networks, we apply weighted link prediction algorithms to predict its possible missing links, as well as to identify potential spurious edges. By cross-checking these links against the other networks, we reconstruct the original network to improve its quality. Then we integrate the six reconstructed networks to get the final integrated network (FINet). We perform network-based disease gene prediction and apply leave-one-out cross validation to assess the quality of the reconstructed networks. At last, to evaluate the applicability of our FINet, we respectively use it and the four meta-networks as background network to conduct obesity associated gene prediction.

## Methods

### Network data sets

In this study, we used six gene association networks of *Homo Sapiens* constructed from publicly obtained data sets as follows.

(i). hsaPPI: a high-quality physical interaction network of human proteins constructed by combining biochemical fractionation data with spectrometric profiling and computational filtering data, in which the weight represents interaction confidence score [31].
(ii). Corum: a protein-protein interaction network of component protein in human protein complexes extracted from the CORUM database [32]. We here used the network constructed in our previous study [33]. The weight represents the number of shared complexes.
(iii). HumanNet: a genome-scale functional association network of human genes which were integrated from 21 large-scale genomics and proteomics data sets. The weight stands for the evidence value used to identify each interaction [34].
(iv). String: a gene association network constructed from the version 9.1 of SRING database [25]. The interaction includes both physical and functional interactions from diverse sources and the weight of each link represents a probabilistic confidence score.
(v). FunCoup: a genome-wide functional coupling (or associations) network constructed from the version 3.0 of FunCoup database [35], which is an integration of huge amounts of genomic data by an optimized Bayesian approach. The weight denotes the confidence score of each association pair.
(vi). FLN: a comprehensive weighted genome-scale network by integrating 16 functional genomics features assembled from 32 sub-features from 6 model organisms, in which nodes represent genes, and edge weights the likelihood that the linked nodes participate in a common biological process [26].
(vii). GO: a weighted gene associated network constructed from the Gene Ontology (GO) database [36] downloaded on March 18, 2015. Two genes are linked in the network if they share at least one GO term. In order to enrich our validation, we finally take links which share at least 3 GO terms, and the number of shared terms is assigned as weight of current link. Data from all the three parts of GO, i.e., biological process, cellular component and molecular function are used. The resulting network contains 9,803,423 gene pairs covering 18,040 human genes.

See Table 1 for the basic information of the six networks. Network (i) to (vi) are used for link prediction and network integration. Since these data sets use different code systems for genes, we first converted their code systems to a unified code system, Entrez gene code. What’s more, the link weights of the data sets vary in different areas. Thus we normalized the weights into the area (0, 1].

**Table 1:** Basic information of the six weighted human gene association networks

Network	hsaPPI	Corum	HumanNet	String	FunCoup	FLN
#Nodes	2,821	2,314	16,243	18,138	16,626	21,657
#Edges	13,880	34,146	476,399	2,165,537	4,044,929	22,388,609
Range of confidence score	0.75~1	1~29	0.4055~4.2569	150~999	0.100~1	0.043~19.032
Range of normalized weight	0.75~1	0.0345~1	0.0953~1	0.1502~1	0.100~1	0.0022~1
Average degree	9.84	29.51	58.66	238.79	486.58	2067.56
Average clustering coefficient	0.169	0.747	0.246	0.232	0.438	0.493

The network (vii), GO, is used to evaluate the performance of our methods. There are 17,797 common genes between GO and the union of the other six networks, reaching 79.72% of the total genes.

### The workflow for the construction of network

**Fig. 1:**
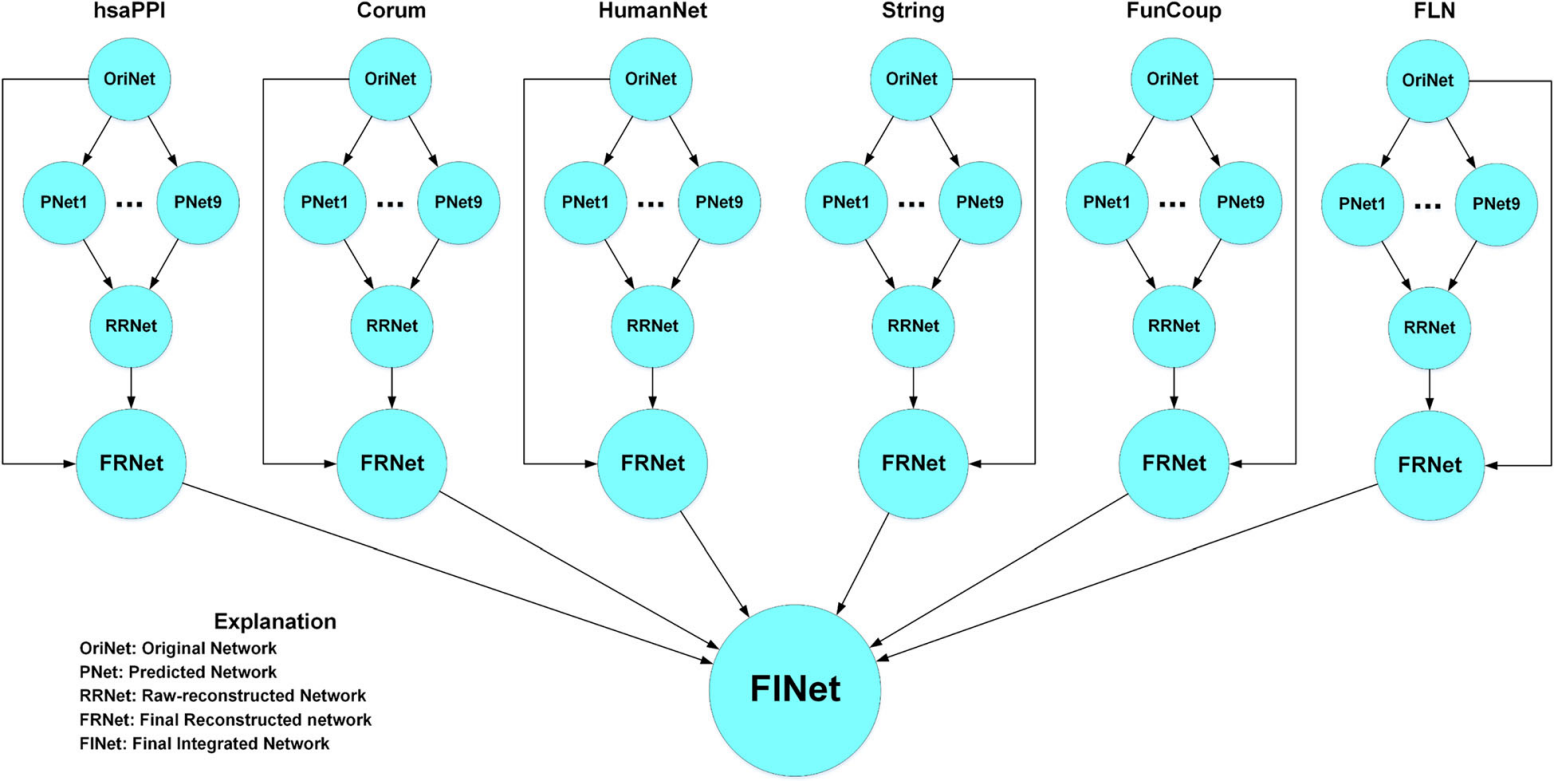
The workflow of network construction from 6 existing networks

In Fig. 1 we simply illustrate our workflow for the construction of a weighted human gene association network from the existing 6 networks, hsaPPI, Corum, HumanNet, String, FunCoup and FLN.

First, we collected source data and constructed the six original networks (OriNet). Second, we performed link prediction in each original network by different similarity-based algorithms to get 9 predicted networks (PNet). Third, for each network, we integrated its 9 predicted networks to obtain a raw-reconstructed network (RRNet). Fourth, for each network, we got a final reconstructed network (FRNet) according to the original network and raw-reconstructed network. At last, we integrated these final reconstructed networks to get the final integrated network (FINet).

### Link and weight prediction

We conducted link prediction and network reconstruction for each of the six gene association networks. They are simple undirected weighted networks, where the weight is a confidence score of the association, representing the probability that the association exists. Thus the weight can be regarded as a symmetric similarity score measuring similarities or affinities between a pair of nodes. Larger similarity weights indicate closer interplays between nodes. Therefore, similarity-based link prediction methods are suitable to be applied for discovering the most possible missing links in these gene association networks.

In a typical similarity-based algorithm, for each pair of nodes *x* and *y*, a similarity score *s*_*xy*_ is assigned to weigh their topological similarity. A higher similarity score corresponds to a higher probability of forming a link between the two nodes.

A lot of similarity indices have been defined based on local [37, 38], global [39, 40] and quasi-local [41] topological information of un-weighted networks respectively, some of which have been extended to weighted networks [42–46]. Considering the large size of network FLN and FunCoup, we focused on local and quasi-local weighted similarity indices.

Local similarity indices only consider the common neighbors of two nodes. Here we used weighted version of Common Neighbors (CN), Adamic-Adar (AA) and Resource Allocation (RA) indices [43, 47], as well as reliable-route weighted similarity indices [33] we defined previously. These similarity indices are listed as follows:

1. Weighted CN index (WCN):

1 $$ {s}_{xy}^{WCN}={\displaystyle \sum_{z\in {O}_{xy}}{w}_{xz}+{w}_{z y}}, $$

(2) Weighted RA index (WRA):

2 $$ {s}_{xy}^{WRA}={\displaystyle \sum_{z\in {O}_{xy}}\frac{w_{xz}+{w}_{z y}}{s_z}}, $$

(3) Weighted AA index (WAA):

3 $$ {s}_{xy}^{WAA}={\displaystyle \sum_{z\in {O}_{xy}}\frac{w_{xz}+{w}_{z y}}{ \log \left(1+{s}_z\right)}}, $$

(4) Reliable-route weighted CN index (rWCN):

4 $$ {s}_{xy}^{rWCN}={\displaystyle \sum_{z\in {O}_{xy}}{w}_{xz}\cdot {w}_{z y}}, $$

(5) Reliable-route weighted RA index (rWRA):

5 $$ {s}_{xy}^{rWRA}={\displaystyle \sum_{z\in {O}_{xy}}\frac{w_{xz}\cdot {w}_{z y}}{s_z}}, $$

(6) Reliable-route weighted AA index (rWAA):

6 $$ {s}_{xy}^{rWAA}={\displaystyle \sum_{z\in {O}_{xy}}\frac{w_{xz}\cdot {w}_{z y}}{ \log \left(1+{s}_z\right)}}. $$

Where *O*_*xy*_ represents the common neighbors set of nodes *x* and *y*, *w*_*xy*_ weighs the link between nodes *x* and *y*, *s*_*z*_ denotes the strength of node *z* defined as the sum of weights for edges link to *z*.

Quasi-local similarity indices not only consider the common neighbors of two nodes, but also take local paths between them into account. Based on the idea of reliable-route weighted similarity indices which measures the similarity of a pair of unconnected nodes by the product of weights of local paths connecting them, we proposed weighted reliable local path similarity indices as follows:

(7) Weighted reliable local path CN index (rWCNLP):

7 $$ {s}_{xy}^{rWCNLP}={\displaystyle \sum_{z\in {O}_{xy}}{w}_{xz}\cdot {w}_{z y}}+\alpha {\displaystyle \sum_{m\in \varGamma (x), n\in \varGamma (y)}{w}_{xm}\cdot {w}_{m n}\cdot {w}_{ny}}, $$

(8) Weighted reliable local path RA index (rWRALP):

8 $$ {s}_{xy}^{rWRALP}={\displaystyle \sum_{z\in {O}_{xy}}\frac{w_{xz}\cdot {w}_{z y}}{s_z}}+\alpha {\displaystyle \sum_{m\in \varGamma (x), n\in \varGamma (y)}{w}_{xm}\cdot {w}_{m n}\cdot {w}_{ny}}, $$

(9) Weighted reliable local path AA index (rWAALP):

9 $$ {s}_{xy}^{rWAALP}={\displaystyle \sum_{z\in {O}_{xy}}\frac{w_{xz}\cdot {w}_{z y}}{ \log \left(1+{s}_z\right)}}+\alpha {\displaystyle \sum_{m\in \varGamma (x), n\in \varGamma (y)}{w}_{xm}\cdot {w}_{m n}\cdot {w}_{ny}}. $$

Where Γ(*x*) is the neighbor set of node *x*, and *α* is a parameter to adjust the contribution of length-3 paths. We here took *α* as 0.001 for hsaPPI and 0.0001 for Corum, HumanNet, String, FunCoup and FLN to penalize the length-3 path. The details for the adjustment of the parameter *α* are shown in Additional file 1: Figure S1.

The similarity score can be regarded as the predicted edge weight, which needs to be normalized with the goal of comparing with the original weight and preparing for network integration. In this study, the similarity score was normalized by Eq. (10) as follows:

10 $$ {w}_{norm}={e}^{-\frac{1}{w}}. $$

### The establishment of raw reconstructed networks

For each of the six networks, we first applied the 9 different similarity indices as defined in eq. (1) ~ (9) to calculate similarity scores, respectively. All possible node pairs were sorted according to their scores in descending order. Then we picked out the same number of node pairs with the highest ranks as in the original network and used these links to construct a new predicted network based on the result of each prediction method, respectively. In this way, we constructed 9 weighted networks for each of the six original gene association networks, respectively. At last, we used 3 steps as follows to integrate all the 9 predicted networks and created a raw reconstructed network.

1. Combine all edges in the 9 predicted networks to obtain an edge union set.
2. For each edge in the union set, integrate the edge weight of different predicted networks to get a topological score.
3. Sort the edges in the union set according to their topological scores in descending order. Then pick out the same number of edges with the highest ranks as in the original network to build a raw-reconstructed network.

In the 2^nd^ step, for each edge in the union set of one original network, we calculated its topological score by integrating normalized similarity scores from 9 different methods as follows,

11 $$ w={\displaystyle \sum_{i=1}^9{\alpha}_i{w}_i}, $$

where *w*_*i*_ is the normalized similarity score from the *i*th link prediction method for one original network, *α*_*i*_ (*i* =1, 2, …9) are parameters that weigh the importance of each prediction method. Here we simply took *α*_*i*_ as $\frac{1}{9}$ to equally weigh their importance.

### The establishment of final reconstructed networks

For each of the six networks, we used the following 4 steps to validate its links in the original network and the raw-reconstructed network so as to create a final reconstructed network.

1. Combine the raw reconstructed network with its original network. As shown in Fig. 2, the links can be classified into 3 groups. We call the links in the original but not in the raw-reconstructed network as *Old* links, those in both the original and the raw-reconstructed network as *Confirmed* links, and the links in the raw-reconstructed but not in the original network as *New* links.

**Fig. 2:**
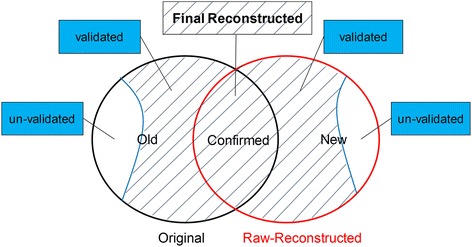
Illustration of our process to get the final reconstructed network for each network
2. Validate the *Old* and *New* links against a test set. The test set is a union of links from all the other 5 original networks and their raw-reconstructed networks, as well as network GO. We think a link as validated if it can be found in the test set.
3. Combine *Confirmed* links with validated *Old* and *New* links to build the final reconstructed network. Thus each link in the final reconstructed network has at least two evidences for its existence. See Fig. 2 for illustration of this process.
4. Compute link weights of the final reconstructed network by integrating topological scores with original link weights of the network.

In the 4^th^ step, we computed link weights of the final reconstructed network by integrating topological scores with original link weights of the network. For a node pair, we define its functional similarity score (*S*_*FS*_) as the weight in original network, and its topological similarity score (*S*_*TS*_) as the score obtained in the 2^nd^ step of creating raw reconstructed network. Thus the functional similarity score for links in the *New* set is zero. Then link weights of the final reconstructed network were computed as follows,

12 $$ S=\beta {S}_{FS}+\left(1-\beta \right){S}_{TS}, $$

where *β* is a parameter to weigh significance of the two kinds of scores. We set *β* as 0.5 to avoid universal larger weights than original links.

### The integration of the 6 final reconstructed networks

We combined all edges in the 6 final reconstructed networks to create the integrated network. The link weight of this network is defined as follows,

13 $$ s=1-\sqrt[6]{{\displaystyle \prod_{i=1}^6\left(1-{S}_i\right)}}, $$

where *S*_*i*_ (*i* = 1, 2, …, 6) represents the link weight in *i*th final reconstructed network, while *S*_*i*_ = 0 if the current network does not include the link. This equation ensures equal roles of the six networks and avoids too small of its second item.

### Performance assessment for link and weight prediction

To assess the quality of 6 raw reconstructed networks created by link and weight prediction, we used links in the GO network as a test set to validate the results. We also performed cross-validation [48] among the 6 networks. Specifically, for each original network, we generated two test sets TONet and TRNet for it. The TONet is a combination of all links in the other 5 original networks, while the TRNet includes all links in the other 5 raw reconstructed networks.

Here we mainly used *precision* [49, 50] to evaluate our networks’ reconstruction and integration. This measure can be calculated as:

14 $$ precision=\frac{TP}{TP+ FP}, $$

where *TP* is the number of network links obtained by the method that also appear in the test network, *FP* is the number of network links obtained by the method that don’t appear in the test network.

Moreover, we calculated the Pearson correlation coefficient (PC) and the mean-squared error (MSE) between the vectors of predicted and original weights for links both in original networks and predicted networks to measure the accuracy of weight prediction. The definition comes as follows:

15 $$ \mathrm{P}\mathrm{C}=\frac{{\displaystyle \sum_{\left( i, j\right)\in L}\left({w}_{i j}-\overline{w}\right)\left({r}_{i j}-\overline{r}\right)}}{\sqrt{\left({\displaystyle {\sum}_{i, j}{\left({w}_{i j}-\overline{w}\right)}^2}\right)\left({\displaystyle {\sum}_{i, j}{\left({r}_{i j}-\overline{r}\right)}^2}\right)}}, $$

16 $$ \mathrm{M}\mathrm{S}\mathrm{E}=\frac{{\displaystyle {\sum}_{i, j}{\left({r}_{i j}-{w}_{i j}\right)}^2}}{N}, $$

where *L* is the set of links both in the original networks and predicted networks, *N* is the number of links in *L*, *w*_*ij*_ is predicted weights for *L*, *r*_*ij*_ is original weights for *L*, $\overline{w}$ and $\overline{r}$ are the corresponding mean value.

### Network-based disease gene prediction

To test the reliability of our methods and the quality of the reconstructed networks, we applied the reconstructed networks in the prediction of disease genes. Given a disease, its known disease genes were used as seed genes, and then candidate genes could be ranked based on their association with these seed genes in the network [51, 52].

We assembled two sets of disease genes for the assessment. The first set includes 1197 distinct disease genes corresponding to 110 different diseases. This set was obtained from the supplementary of ref. [26] and the disease gene symbol was mapped into its entrez ID. The second set only includes obesity related genes, in which 24 genes were extracted from the OMIM database [53] and other 373 genes were collected from a literature [54] (see Additional file 1).

For a particular disease and its seed genes, the association of each candidate gene *i* with the disease is quantified by a score as follows:

17 $$ {S}_i^{DA}={\displaystyle \sum_{j\in seeds}{W}_{i j}}, $$

where *S*_*i*_^*DA*^ is the disease association score, and *w*_*ij*_ is the edge weight connecting gene *i* and seed *j*. The score, thereby, will be 0 if the gene is not connected with any seeds.

To assess the overall performance of a reconstructed network in disease gene prediction, we conducted leave-one-out cross validation using the first disease gene set. For each disease, each known disease gene was taken out as a test gene, and the remaining disease genes were used as seeds. Then each gene in the network was assigned a disease association score *S*_*i*_^*DA*^ based on its proximity to the seeds and each test gene was ranked among all genes in the network. We further pooled together all genes and calculated the precision as the fraction of disease genes above the cutoff at various rank cutoffs. A larger fraction suggests a better performance for the current network. At last, we conducted a case study on obesity gene prediction. The 24 obesity genes from the OMIM database were used as seeds and the other 373 genes reported in the literature were test genes.

## Results and Discussion

### Comparison of the six original weighted human gene association networks

Among the 6 networks under study, hsaPPI and Corum have much smaller sizes or scales than the other four networks. This is because they are high-confidence protein-protein interaction networks of human beings which include specific molecular interaction information between proteins. Specifically, the network hsaPPI is constructed from the experimental biochemical co-fractionation data in consistence with information from curated public databases and literatures. The network Corum was constructed to represent theoretical links between component proteins of experimentally validated protein complexes, which represents a specific class of high-confidence protein-protein interactions, i.e., co-complex memberships. In contrast, the other four networks, HumanNet, String, FunCoup and FLN, are from meta-databases constructed by integrating both physical and functional interactions between human genes available from numerous sources of different features and using their own scoring systems to weigh the confidence of each association. They include much more general gene association information, but at the same time, they are noisier.

**Fig. 3:**
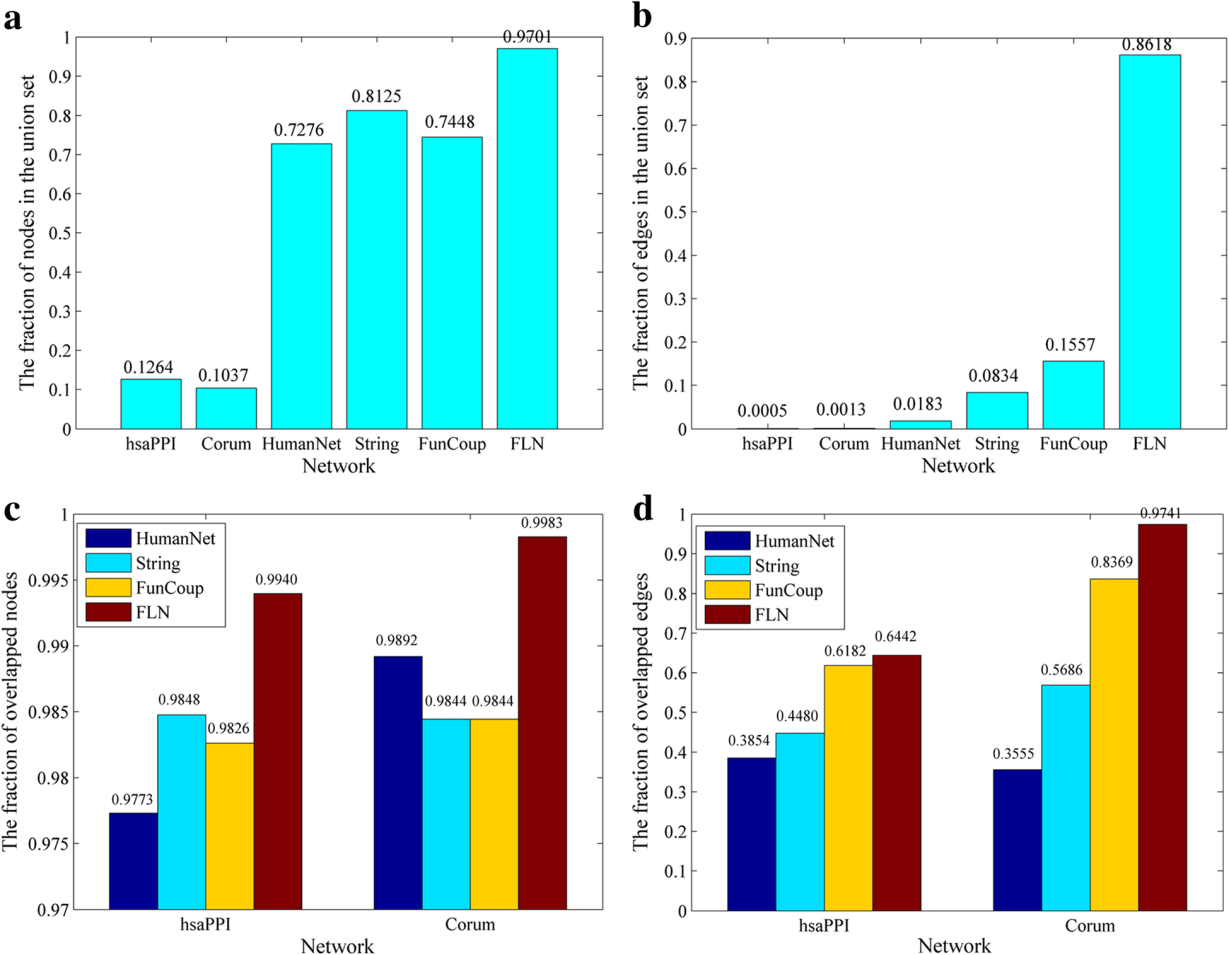
The comparison of nodes and edges in all original networks. **a**, **b** the fractions of nodes and edges for each network in the union sets. **c**, **d** the fractions of overlapped nodes and edges of the two small networks (hsaPPI and Corum) with the other four large networks

Simply combining all the nodes and edges of the 6 networks, we obtain complete node and edge sets that include 22,324 distinct genes and 25,978,000 different association relationships respectively. The network consisting of the complete node and edge sets is called original union network (OUNet). We compare the sizes and overlapping extents of these networks by mapping each network’s nodes and edges to the node and edge set of OUNet, respectively. As shown in Fig. 3a, the complete node set includes almost all nodes of FLN (97%) and most nodes of HumanNet (73%), String (81%) and FunCoup (74%), suggesting these meta-networks have a large fraction of common genes. Figure 3b implies that the fraction of edges in the complete edge set is approximately proportional to the number of edges of each network.

Ideally, since hsaPPI and Corum are high-confidence protein-protein interaction networks, almost all of their nodes and edges should be included in the four large networks from meta-databases. However, Fig. 3c and d show that the speculation is true for nodes but not for edges. The four meta-networks include almost all nodes of the two specific networks. However, considerable part of edges in the two specific networks do not appear in the four meta-networks, while the fractions of the two specific networks’ edges in the four meta-networks are positively correlated with the number of edges in the meta-networks.

**Fig. 4:**
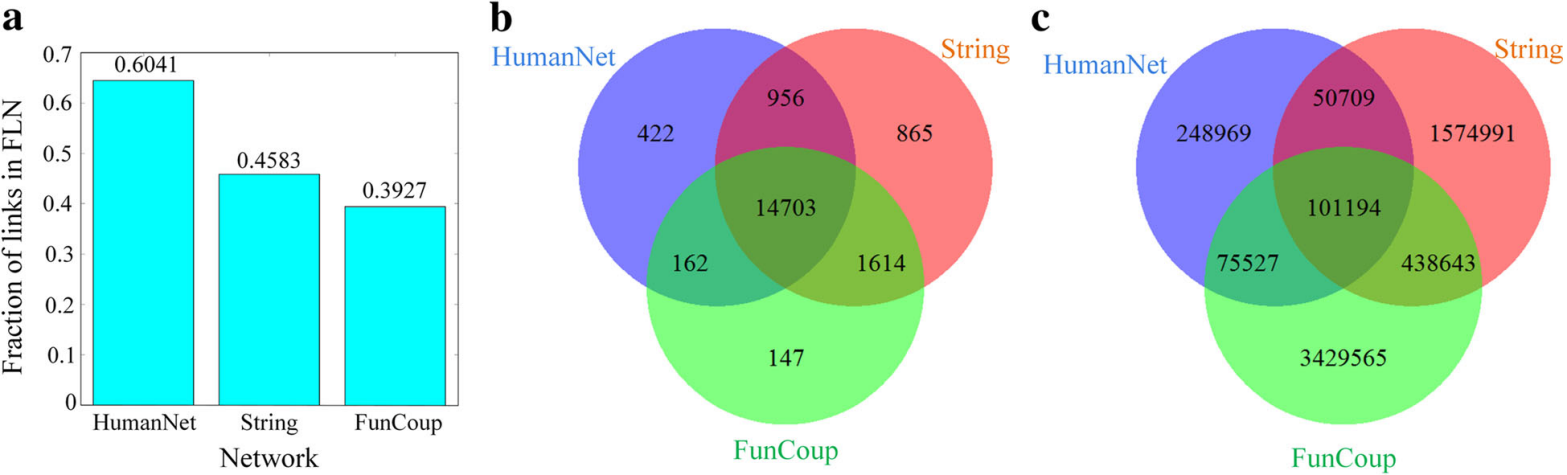
The comparison of the first three meta-networks, HumanNet, String, and FunCoup. **a** the fractions of edges included in FLN. **b**, **c** the number of overlapped nodes and edges among these three meta-networks

Comparing edges of the four meta-networks (HumanNet, String, FunCoup and FLN), we found that although the network FLN is much larger than the other three networks, it does not contain most edges of the first three networks (Fig. 4a). Considering FLN’s super large size of edges which may cause considerable differences in magnitude, we further compared the first three meta-networks. As shown in Fig. 4b and c, they have 14703 and 101194 common nodes and edges, respectively. Although the three networks have a large fraction of common nodes (taking 90.52%, 81.06% and 88.43% of the total in HumanNet, String, and FunCoup, respectively), there is quite limited fraction of common edges (only taking 14.35%, 3.16% and 1.69% of the total in HumanNet, String, and FunCoup, respectively).

The comparisons suggest that it is necessary to integrate all of these networks to create a network with larger size and higher confidence.

### Network raw-reconstruction and weight prediction

**Fig. 5:**
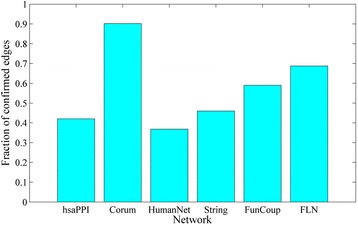
The fraction of confirmed links in the 6 original networks

For each of the six weighted gene association networks, we applied 9 similarity-based link prediction methods to construct 9 different predicted networks, respectively. Then we integrated them to obtain a raw-reconstructed network which owns the same number of edges as the original network. As Fig. 2 shows, links in the union of the original and raw-reconstructed networks can be classified into 3 groups, *Old, New* and *Confirmed.* Figure 5 shows the fraction of confirmed links in the 6 original networks. It can be seen that network Corum, FunCoup and FLN have much larger part of confirmed links than the other 3 networks. This is because that these three networks have much bigger clustering coefficients (See Table 1), thus more links in the original networks get higher similarity scores and rank on the top of the list of node pairs.

To assess the performance of link predictions and evaluate the functional relevance of the raw-reconstructed networks, we compared each raw reconstructed network with its original network against three test sets, TONet, TRNet, and GONet. The 5 groups of links, *i.e.*, links in the raw reconstructed network, the original network, *Old, New* and *Confirmed* set, were used as query sets for evaluation. Cross-validation was conducted by checking the link sets corresponding to one network against two test sets TONet and TRNet, which were constructed by respectively combining all links in the other 5 original networks and the other 5 raw reconstructed networks. Links in the GO network were used as test set for the evaluation of functional relevance. A link in a query set is regarded as validated by one test set if it can be found in this test set.

**Fig. 6:**
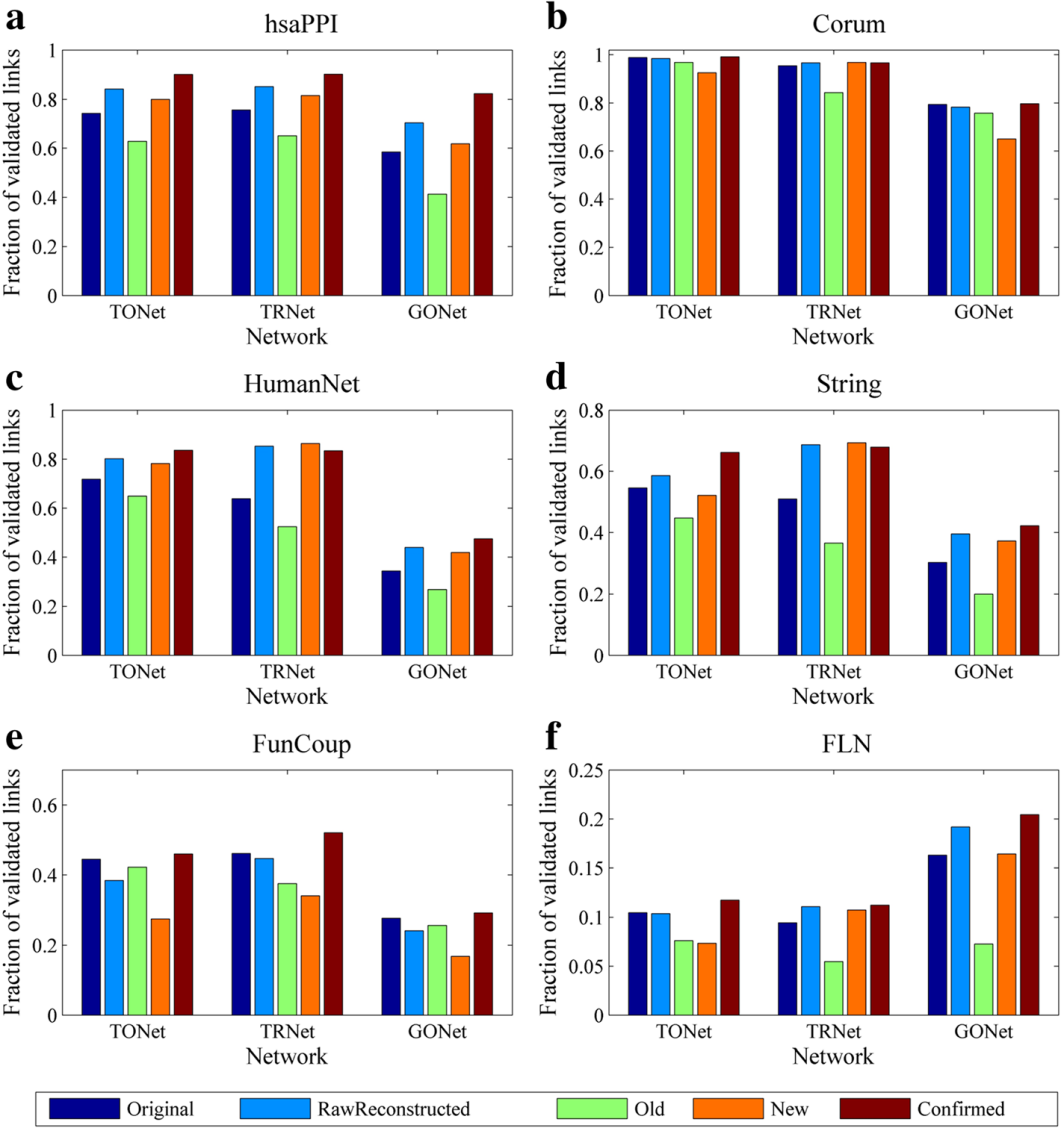
The evaluation of 5 different edge groups against 3 test sets. These 5 edge groups (Original, Raw Reconstructed, Old, New, and Confirmed) correspond to each of the 6 networks ((**a**) hsaPPI; (**b**) Corum; (**c**) HumanNet; (**d**) String; (**e**) FunCoup; (**f**) FLN), and the 3 test sets are TONet, TRNet, and GONet, respectively

As shown in Fig. 6, in most cases, validated links in raw-reconstructed networks and *New* groups are more than or very close to those in corresponding original networks and *Old* groups, respectively. In addition, the confirmed group has the highest percentage of validated links than the other 4 groups of links of the same network. That the fractions of validated links slightly decrease in raw-reconstructed networks of Corum and FunCoup than in their original networks could be due to their higher percentages of confirmed links (see Fig. 6).

These comparisons show that links in raw-reconstructed networks have higher or similar extent of functional relevance than those in corresponding original networks, suggesting that our algorithm could effectively increase the size of the original gene association networks and reduce their noises. It is noted that in some earlier studies, the raw-reconstructed networks obtained from their link prediction algorithms were considered as final de-noised networks [22, 23].

To measure the accuracy of weight prediction, for each of the six networks, we then calculated the Pearson correlation coefficient (PC) and the mean-squared error (MSE) between the vectors of predicted and original weights for links both in raw-reconstructed network and original networks. Table 2 shows that all the Pearson correlation coefficients are larger than zero and all the *p-*values are much smaller than 0.05, indicating the statistically significant positive linear correlation between the weights in all six cases. All the MSE values are rather small, suggesting a high consistence between the normalized scores and weights similar to the Pearson correlation coefficients. That Corum has a minimum MSE, furthermore, indicates its high prediction accuracy.

**Table 2:** The result of weight prediction of five networks

Network	hsaPPI	Corum	HumanNet	String	FunCoup	FLN
MSE	0.0680	0.0508	0.0879	0.1779	0.1631	0.1050
Pearson correlation coefficients	0.5392	0.4070	0.4827	0.4965	0.5798	0.5627
*p-*value	4.82 × 10^−362^	0	0	0	0	0

### Network final reconstruction

For each of the six networks, we combined links in *Confirmed* set with validated links in *Old* and *New* sets to build the final reconstructed network. In this way, links in each final reconstructed network have at least two evidences for existence. These evidences are links from all the other 5 original networks and their raw-reconstructed networks, as well as network GO. We used eq. (12) to get link weights of the final reconstructed network by integrating similarity scores calculated by eq. (11) (called topological scores) with original link weights of the network (called functional scores).

We listed the number of links in the 6 original networks and their final reconstructed networks in Table 3. It can be seen that, by reconstruction, link numbers in the 5 smaller networks get increased, while only the largest network FLN becomes smaller. This is because that the *Old* and *New* groups in the smaller networks are more likely to be found in other networks, which makes these networks enlarge. Similarly, the *Old* and *New* groups in the largest network have small probably to be found in other much smaller networks, which makes this network shrink.

**Table 3:** Number of edges in original and final reconstructed networks

Network	hsaPPI	Corum	HumanNet	String	FunCoup	FLN
# links in OriNet	13,880	34,146	476,399	2,165,537	4,044,929	22,388,609
# links in FRNet	18,846	37,390	664,131	2,560,045	4,057,603	18,178,219
Change of increase percentage	35.78%	9.50%	39.41%	18.22%	0.31%	−18.81%

**Fig. 7:**
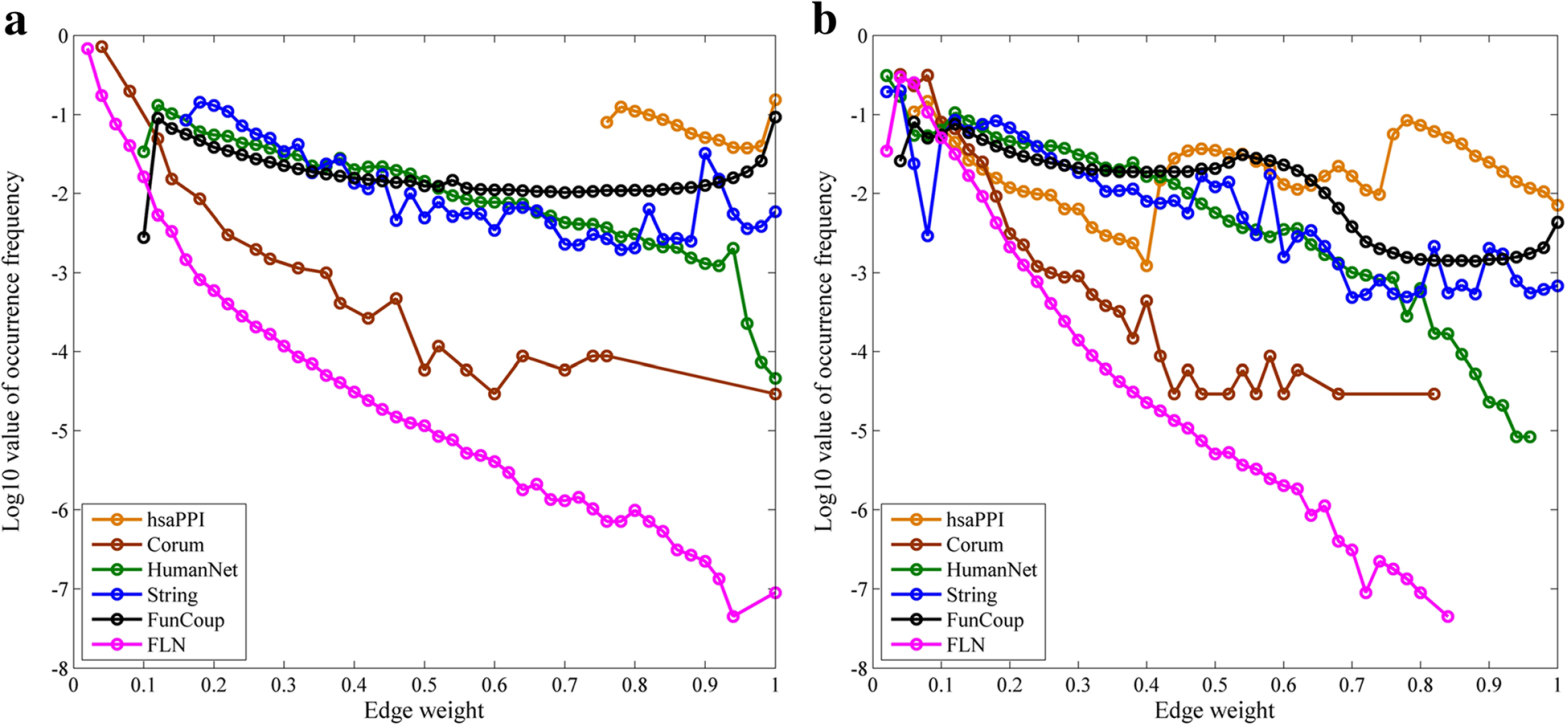
The log10 value of occurrence frequency of edge weights for all original networks and corresponding final reconstructed networks. **a** original networks, (**b**) final reconstructed networks

To explore the change of the link weight distributions in the networks, we depicted the distributions of link weight for the 6 original networks and their final reconstructed networks in Fig. 7. For all the networks, the semi-log scale plots for the distribution functions of link weight are decreasing curves, suggesting that large fractions of edges in these networks own small link weights. That is, only small fractions of gene associations have high confidence scores. Among the six original networks, FLN’s edge weight distribution curve locates the lowest and decreases most sharply. This phenomenon suggests that although network FLN has much more links than the other networks, a great fraction of them has low confidence score. In fact, its link weights of about 90% links are smaller than 0.05. The network hsaPPI only includes high confidence links, in which the lowest confidence score is 0.75. Thus its distribution curve locates the highest. From Fig. 7 (b) we can see that the hsaPPI’s final reconstructed network increases some low-confidence links and still keeps a large fraction of high-confidence links. Figure 7 also shows that the edge weight distribution curves of the final reconstructed networks almost keep the same order and tendency as the original networks. This suggests that our reconstruction did not significantly change the weight distribution features of the networks.

**Fig. 8:**
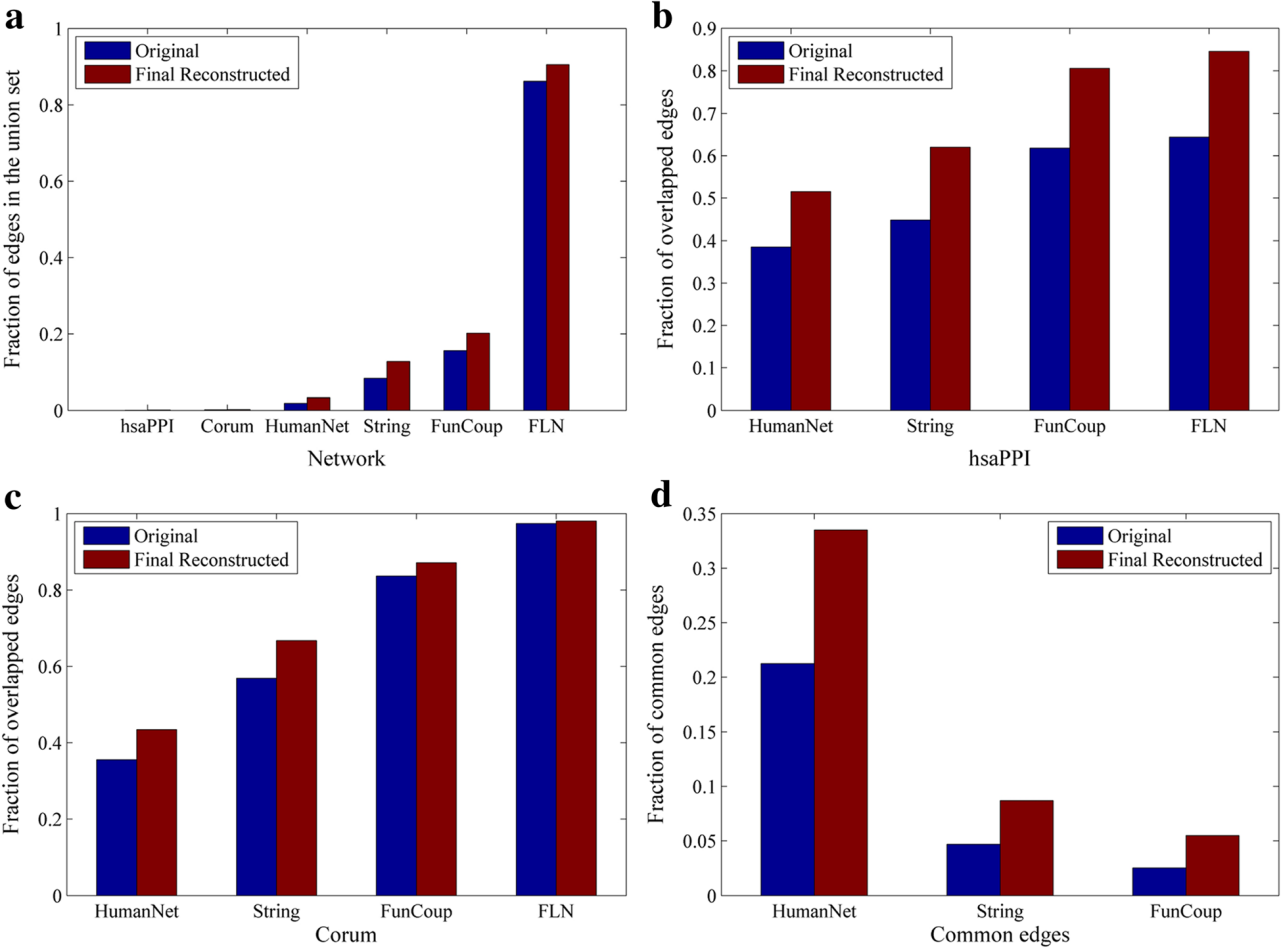
The comparison of edges between original networks and final reconstructed networks. **a** the fraction of edges for each network in corresponding union sets. **b**, **c** the fractions of overlapped edges of the two small networks (hsaPPI and Corum) with the other four large networks. **d** the fraction of common links of the three networks (HumanNet, String and FunCoup)

To see if the final reconstructed networks have higher confidence than their corresponding original networks, we compared edges between original networks and final reconstructed networks from different perspectives (See Fig. 8). By mapping each network’s edges to the union of original/final reconstructed edge set respectively, we found that the fraction in the final reconstructed networks increases obviously (Fig. 8a). As shown in Fig. 8b and c, in all cases, after reconstruction, significantly more edges of the two specific small networks hsaPPI and Corum appear in the four meta-networks. Figure 8d shows that the fractions of common links of the three meta-networks HumanNet, String, and FunCoup have increased significantly in our final reconstructions. These comparisons suggest a higher confidence of our reconstructed network compared with the originals.

In summary, by reconstruction, common information of the 6 final reconstructed networks increase significantly, suggesting the reliabilities of all networks be enhanced compared to their original networks. Therefore, it is reasonable and of great necessary to integrate the 6 final reconstructed networks to get a bigger union network.

### Integration of different final reconstructed networks

We combined the links in the 6 final reconstructed networks to create the final integrated network (FINet) and calculated link weight of this network by eq. (13). This FINet has 20,091,321 links, which is much more than any of the first 5 original networks and slightly less than the biggest network FLN. See the Additional file 2 for the data of FINet.

To verify the functional relevance of the links in the FINet, we compared this network and the union network of original networks (OUNet) with the GO network (GONet). Specifically, we first mapped edges of FINet and OUNet to GONet to identify their overlapped links with GONet, respectively. Then we calculated average shared GO terms of the node pairs corresponding to the overlapped links. As Table 4 shows, compared with the OUNet, the FINet has much more links in GONet, while the average shared GO terms of node pairs corresponding to these links do not decrease significantly. This result suggests that links in the final integrated network exhibit high functional relevance. Therefore, our algorithm could effectively enlarge and de-noise the gene association networks.

**Table 4:** Comparison of OUNet and FINet with GONet

Network	OUNet	FINet
Number of overlapped links	4,255,042	5,400,858
Average shared GO terms of node pairs for overlapped links	4.211	4.133

### Assessment of the reconstructed networks in the context of disease gene prediction

One important application of gene association network is to be used as a background network in the prediction of disease genes [51, 55–60]. This is due to an observation that genes associated with the same disease tend to be close with each other in the network. To assess the reliability of our methods, here we conducted network-based disease gene prediction using different networks as background networks.

First, we tested the performance of our final reconstructed networks (FRNet) of the four meta-networks in the prediction of disease genes. Ref. [23] proposed a random walk with resistance (RWS) algorithm to predict missing links of a network and reconstruct a PPI network by taking out the same number of node pairs with the highest similarity scores as in the original network, which is equivalent to our raw reconstructed network (RRNet). For each meta-network, respectively utilizing the original network (OriNet), its FRNet and RRNet obtained by our method and its RRNet got by RWS algorithm (RWS-RRNet) as background network, we conducted disease gene prediction in these networks. We performed leave-one-out cross validation using our first disease gene set, which includes 1197 distinct disease genes corresponding to 110 different diseases. For each disease, we successively took out one disease gene and used the rest of the genes as input to predict this one. Eq. (17) was applied to calculate a disease association score for each gene in the network and ranked them decreasingly. Finally, we pooled all the test cases together and calculated the fraction of the tested disease genes ranked above various rank cutoffs.

**Fig. 9:**
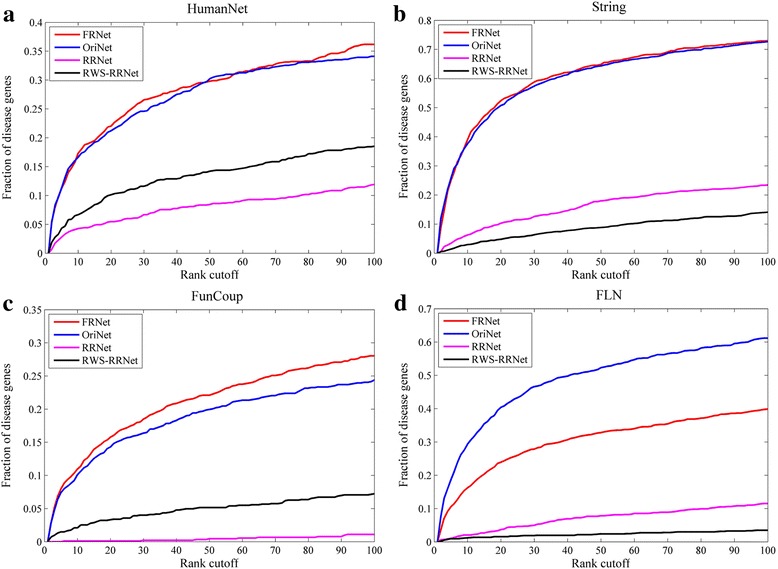
Performance comparison of disease gene prediction based on each meta-network (OriNet), its final reconstructed network (FRNet), raw reconstructed network (RRNet), and its raw reconstructed network got by RWS algorithm (RWS- RRNet). The four meta-networks are (**a**) HumanNet; (**b**) String; (**c**) FunCoup; (**d**) FLN

As shown in Fig. 9, for each of the four meta-networks, its FRNet is significantly superior to both of its corresponding RRNets in disease gene prediction, supporting our strategy that further builds a final reconstructed network from the original and raw reconstructed network by cross-validation. The final reconstructed network rectifies the shortcoming of raw reconstructed network which discards quite large part of original links and adds many new links. For each of the three smaller networks (HumanNet, String and FunCoup), its FRNet also exhibits better performance than the network itself (OriNet), implying that the enlarged network includes more meaningful information. Only exception is that FLN’s FRNet shows poorer performance than itself. We think this is because that the FRNet of this largest network becomes much smaller than the original network, thus much information gets lost. In addition, when comparing the two raw reconstructed networks, it appears that our raw reconstructed networks (RRNet) and RWS-RRNet networks respectively perform better in half of the networks. This suggests that these two different link prediction algorithms have good performance in different networks.

**Fig. 10:**
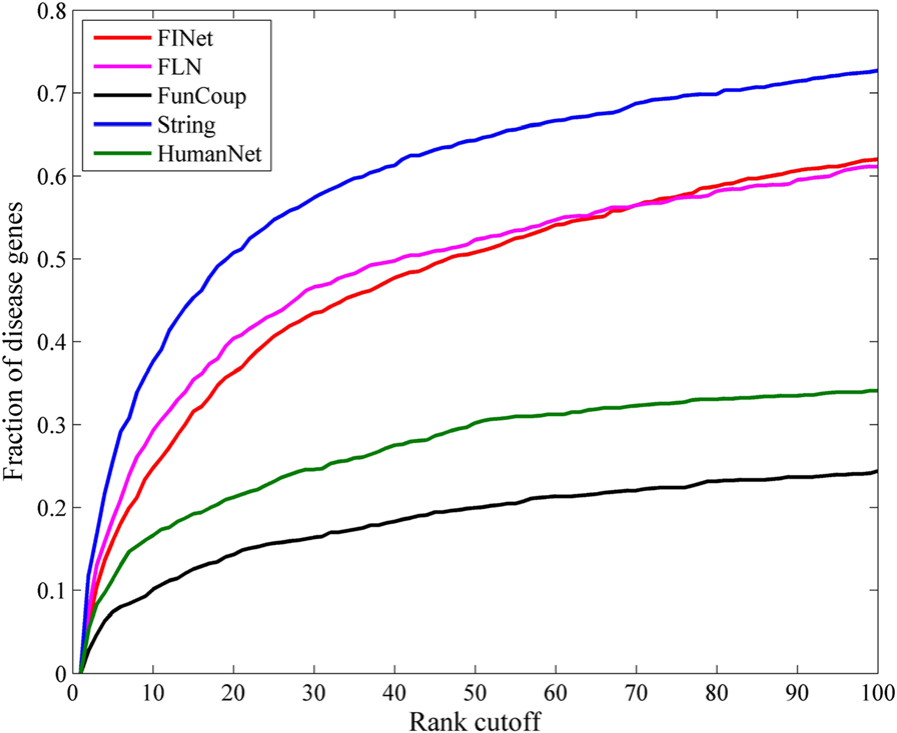
Performance comparison of disease gene prediction based on our final integrated network (FINet) and the four original meta-networks (FLN, FunCoup, String and HumanNet)

Then, similarly, using the same disease gene set and leave-one-out cross validation method, we test the performance of our final integrated network (FINet) in comparison with the four original meta-networks (HumanNet, String, FunCoup and FLN). As Fig. 10 shows, the FINet performs better than HumanNet and FunCoup but poorer than String and FLN. Notably, the performances of FounCoup and HumanNet are much poorer than String and FLN, although the size of FounCoup is about twice of String. Thus we guess that the poorer performance of FINet is probably caused by networks HumanNet and FunCoup, which exhibit poor performance.

**Fig. 11:**
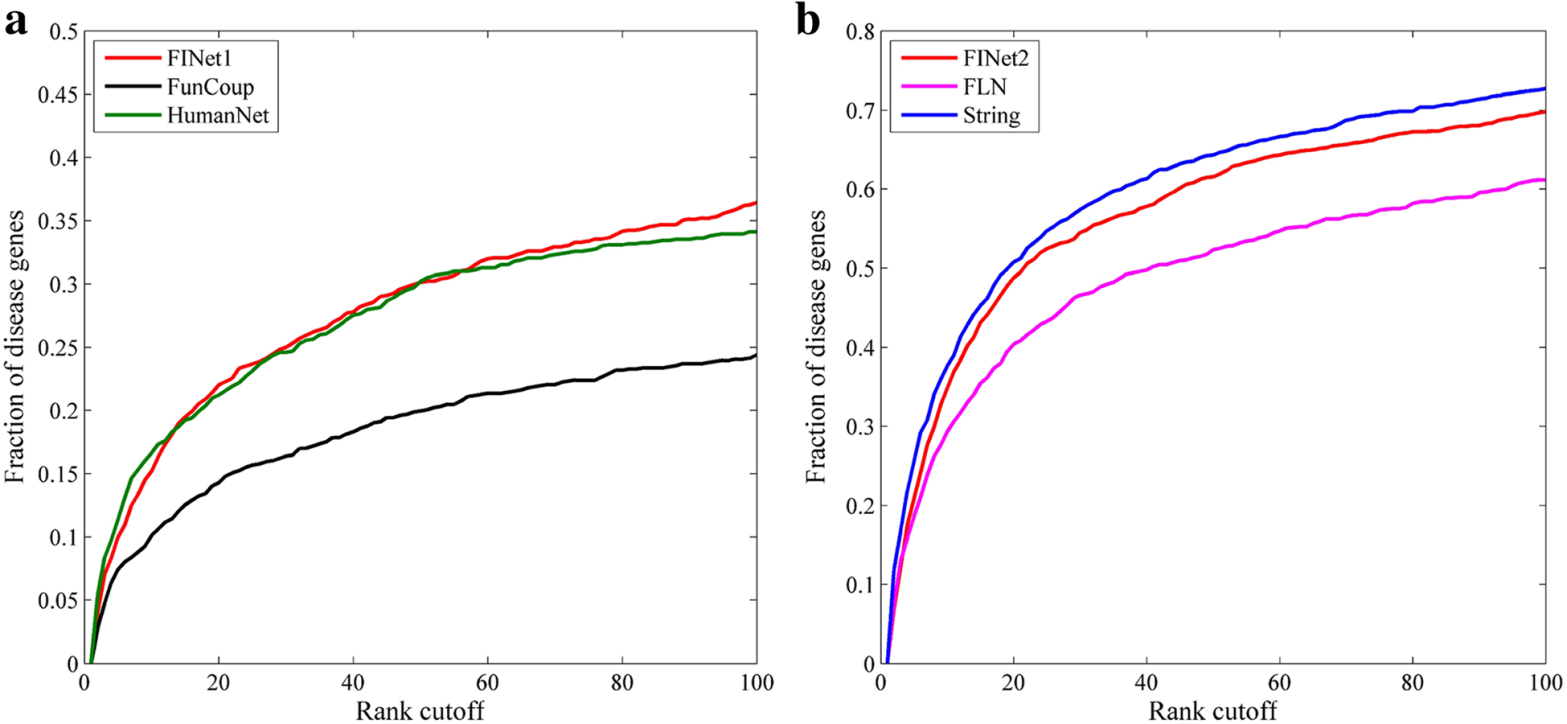
Performance comparison of disease gene prediction based on different final integrated networks and their corresponding original networks. **a** The final integrated network (FINet1) is constructed from String and FLN; (**b**) The final integrated network (FINet2) is constructed from HumanNet and FunCoup

To verify our conjecture, we used our workflow (Fig. 1) to construct two FINet networks by integrating String with FLN, HumanNet with FunCoup and named them as FINet1 and FINet2, respectively. In Fig. 11 we compared the performance of these FINet networks with their corresponding original networks. It shows that the performance of the FINet networks is between the good and the poor component networks and much closer to the good one. These results validate our conjecture and suggest that the performance of the final integrated network could be reduced by networks which have much poorer performance.

### An application of the final integrated network: prediction of obesity associated genes

To further test the effectiveness of the final integrated network, we conducted disease gene prediction in the case of obesity, respectively using FINet and the 4 original meta-networks (HumanNet, String, FunCoup and FLN) as background network. Using the 24 known obesity associated genes from the OMIM as seeds, we applied eq. (17) to predict other disease genes. The other 373 genes from the literature were used as test genes. The numbers of seed and test genes appearing in different background networks are listed in Table 5. Except HumanNet, the other 4 networks include same number of seed genes. However, the number of test genes appearing in these networks is different. FINet and FLN include more test genes than the other networks, suggesting that they are more informative.

**Table 5:** The numbers of seed and test genes in the 5 background networks and validated genes at different cutoff

Network	HumanNet	String	FunCoup	FLN	FINet
# seed genes	22	23	23	23	23
# test genes	351	352	348	356	355
# validated genes at rank cutoff 1	1	0	0	1	1
# validated genes at rank cutoff 30	8	25	3	26	24
# validated genes at rank cutoff 100	21	65	4	63	62
# validated genes at rank cutoff 150	35	79	5	79	83

We treated all genes in the background network as candidate genes and assigned scores to them by eq. (17) for prioritization. Then we ranked the genes in each background network decreasingly according to their scores. Based on the rank, we could predict the top *h* ones as associated with the disease obesity. In Table 5, we listed different prediction results for the test genes with different *h*-values (1, 30, 100, and150). It can be seen that, the first ranked gene is obesity associated gene when applying FINet, FLN and HumanNet as background network. Meanwhile, the numbers of validated disease genes at different rank cutoffs by String, FLN and FINet are similar and much higher than that by HumanNet and FunCoup, suggesting their much better performance than the other two networks.

To further examine the ability of these three networks FINet, FLN and String in identifying unknown disease genes, we checked the possibilities that their un-validated genes in the top 100 ranks are also disease genes. Considering that more records of loci information about one specific gene in the PubMed suggest a higher probability that the gene is associated with diseases, we searched these genes in the PubMed using their gene symbol and “obesity” as keywords. Finally, we got the number of records for collected information about gene loci.

**Fig. 12:**
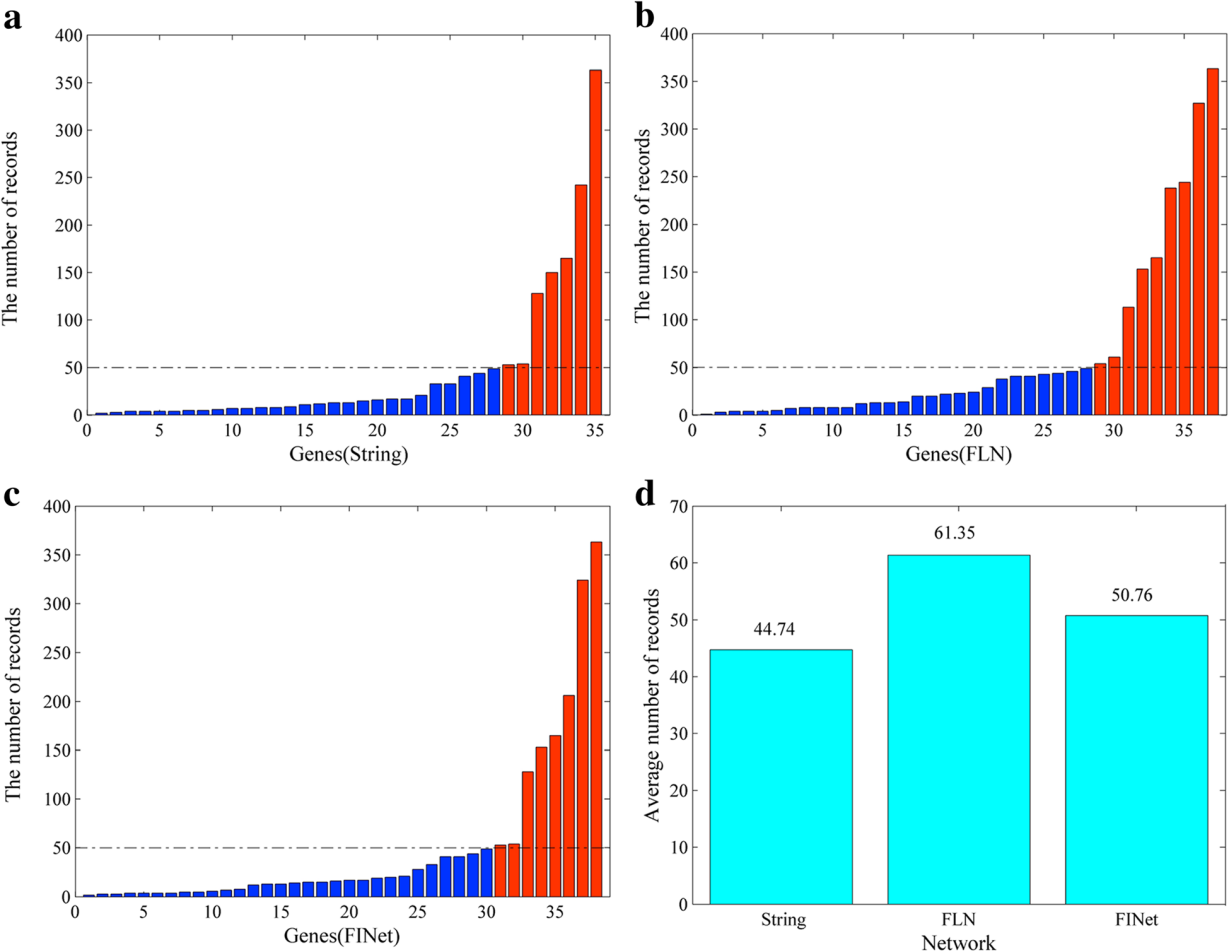
The numbers of records of collected information about gene loci for un-validated genes in PubMed. **a**, **b**, **c** the numbers of records compared to 50 for networks, String, FLN and FinalNet. (Red bars highlights genes with at least 50 records, while blue bars stand for genes with lower than 50 records.) **d** the average number of records

We set number of Pubmed records about a gene’s loci as high record and compared the numbers of the records with 50. We also calculated the average number of all un-validated genes for these three networks. It can be observed from Fig. 12a, b and c that the numbers of genes with at least 50 records are 7, 9, 8 for String, FLN, and FINet, respectively. The average number of records for FINet is higher than that for String and lower than FLN (Fig. 12d). These results imply that FINet holds a better performance for identifying unknown disease genes than String, suggesting its high practicability and effectiveness.

## Conclusion

To expand and de-noise the known human gene association data, we proposed a workflow to construct a weighted human gene association network (named FINet) based on six existing networks, hsaPPI, Corum, HumanNet, String, FunCoup and FLN. First, for each network, we conducted link prediction to predict its possible missing links and identify potential spurious edges using 9 weighted similarity indices. We then combined the 9 link prediction results to obtain a raw-reconstructed network. By cross-checking the links in original and raw-reconstructed networks against the other networks and the test network GONet constructed from GO database, we next built a final reconstructed network for each network. At last, all final reconstructed networks were integrated to construct a final integrated network (FINet). To validate its applicability, we utilized this network as background network to conduct disease associated genes prediction.

This FINet has much more links than any of the first 5 original networks and slightly less links than the largest network FLN. Thus we have enlarged most of the original networks. Compared with original networks, the common information among the final reconstructed networks increase notably, suggesting that the final reconstructed networks are of better reliability. Mapping links in the final integrated network to GO confirms their high functional relevance. In addition, our final integrated network presents good performance in disease gene prediction, which indicates its reliability and application significance. Our workflow presented here could be an insightful framework for integrating and refining existing gene association data.

## Additional files

Additional file 1: The Integration of Weighted Human Gene Association Networks Based on Link Prediction. **Table S1**. The seed genes associated with obesity obtained from OMIM. **Table S2**. The test genes for obesity associated genes prediction. **Figure S1**. The adjustment of parameter α in quasi-local similarity indices by link prediction accuracy measured by precision. (DOCX 57 kb)
Additional file 2: data of our final integrated network FINet. (TXT 374277 kb)

## Abbreviations

BIND: The biomolecular interaction network database
FINet: The final integrated network
FRNet: The final reconstructed network
GONet: The GO network
HPRD: Human protein reference database
MSE: The mean-squared error
OriNet: The original network
OUNet: Original union network
PC: The Pearson correlation coefficient
PNet: The predicted network
RRNet: The raw-reconstructed network
rWAA: Reliable-route weighted Adamic-Adar index
rWAALP: Weighted reliable local path Adamic-Adar index
rWCN: Reliable-route weighted common neighbors index
rWCNLP: Weighted reliable local path common neighbors index
rWRA: Reliable-route weighted resource allocation index
rWRALP: Weighted reliable local path resource allocation index
WAA: Weighted Adamic-Adar index
WCN: Weighted common neighbors index
WRA: Weighted resource allocation index
