# Supplementary material for: The integration of weighted human gene association networks based on link prediction
Source: BMC Syst Biol. 2017 Jan 31;11:12. doi: 10.1186/s12918-017-0398-0 (PMC5282786; doi:10.1186/s12918-017-0398-0)
Supplement: Additional file 1: — The Integration of Weighted Human Gene Association Networks Based on Link Prediction. Table S1. The seed genes associated with obesity obtained from OMIM. Table S2. The test genes for obesity associated genes prediction. Figure S1. The adjustment of parameter α in quasi-local similarity indices by link prediction accuracy measured by precision. (DOCX 57 kb) [file 12918_2017_398_MOESM1_ESM.docx]

# Additional file for “The Integration of Weighted Human Gene Association Networks Based on Link Prediction”

## Contents

**Table S1** The seed genes associated with obesity obtained from OMIM.

**Table S2** The validation genes for obesity associated genes prediction.

**Figure S1** The adjustment of parameter α in quasi-local similarity indices by link prediction accuracy measured by precision

## Table S1 The seed genes associated with obesity obtained from OMIM.

| **Gene Symbol** | **OMIN ID** | **Entrez ID** |
| --- | --- | --- |
| ADRB2 | 109690 | 154 |
| ADRB3 | 109691 | 155 |
| AGRP | 602311 | 181 |
| AKR1C2 | 600450 | 1646 |
| LEP | 164160 | 3952 |
| LEPR | 601007 | 3953 |
| MC3R | 155540 | 4159 |
| MC4R | 155541 | 4160 |
| NTRK2 | 600456 | 4915 |
| PCSK1 | 162150 | 5122 |
| ENPP1 | 173335 | 5167 |
| POMC | 176830 | 5443 |
| PPARG | 601487 | 5468 |
| SIM1 | 603128 | 6492 |
| UCP1 | 113730 | 7350 |
| UCP2 | 601693 | 7351 |
| UCP3 | 602044 | 7352 |
| NR0B2 | 604630 | 8431 |
| SDC3 | 186357 | 9672 |
| SLC6A14 | 300444 | 11254 |
| GHRL | 605353 | 51738 |
| FTO | 610966 | 79068 |
| PPARGC1B | 608886 | 133522 |
| DDH2 | 615612 | 102775567 |

## Table S2 The validation genes for obesity associated genes prediction.

| **Gene Symbol** | **Entrez ID** | **Gene Symbol** | **Entrez ID** | **Gene Symbol** | **Entrez ID** | **Gene Symbol** | **Entrez ID** |
| --- | --- | --- | --- | --- | --- | --- | --- |
| ASIP | 434 | CRH | 1392 | IKBKB | 3551 | PYY | 5697 |
| ABCC8 | 6833 | CRHR1 | 1394 | IL1A | 3552 | PRKAA2 | 5563 |
| ABCG5 | 64240 | CRHR2 | 1395 | IL1RN | 3557 | PRKACA | 5566 |
| ACACB | 32 | CYP19A1 | 1588 | IL6 | 3569 | PRKACB | 5567 |
| ADIPOQ | 9370 | CYP7A1 | 1581 | IL6R | 3570 | PRKAB1 (aka AMPK) | 5564 |
| ACACA | 31 | DBH | 1621 | INS | 3630 | PRKAG3 | 53632 |
| ACE | 1636 | DBI | 1622 | INSIG1 | 3638 | PRKAR1A | 5573 |
| ACP1 | 52 | DDIT3 | 1649 | INSR | 3643 | PRKAR2B | 5577 |
| PHACS | 84680 | POU3F4 | 5456 | IPW | 3653 | PRKCQ | 5588 |
| ADA | 100 | DGAT1 | 8694 | IRS1 | 3667 | PRLR | 5618 |
| ADCYAP1 | 116 | DGAT2 | 84649 | IRS2 | 8660 | PROP1 | 5626 |
| ADD1 | 118 | DHCR24 | 1718 | JUN | 3725 | PTGIR | 5739 |
| ADIPOR1 | 51094 | DIO2 | 1734 | KCNA3 | 3738 | PTGER1 | 5731 |
| ADIPOR2 | 79602 | DLK1 | 8788 | KCNJ11 | 3767 | PTGER3 | 5733 |
| ADORA1 | 134 | DRD2 | 1813 | KL | 9365 | PTGER4 | 5734 |
| ADORA2A | 135 | DRD3 | 1814 | LDLR | 3949 | PTGFR | 5737 |
| ADORA2B | 136 | DRD4 | 1815 | LEP | 3952 | PTGS2 | 5743 |
| ADRA1B | 147 | EIF4EBP1 | 1978 | LEPR | 3953 | PTPN1 | 5770 |
| ADRA2A | 150 | ENPP1 | 5167 | LHB | 3972 | PTPRF | 5792 |
| ADRA2B | 151 | ESR1 | 2099 | LIFR | 3977 | PTTG1 | 9232 |
| ADRB1 | 153 | ESRRA | 2101 | LIPC | 3990 | PWCR1 | 63968 |
| ADRB2 | 154 | ETS1 | 2113 | LIPE | 3991 | RAB3GAP1 | 22930 |
| ADRB3 | 155 | FABP2 | 2169 | LMNA | 4000 | RAPTOR | 57521 |
| ADFP | 123 | FABP4 | 2167 | LPIN1 | 23175 | RARA | 5914 |
| AEBP1 | 165 | CFD | 1675 | LPL | 4023 | RARG | 5916 |
| AGPAT1 | 10554 | FASN | 2194 | LRPAP1 | 4043 | RASGRF1 | 5923 |
| AGPAT2 | 10555 | FGF1 | 2246 | ITGAM | 3684 | RB1 | 5925 |
| AGRP | 181 | FGFR3 | 2261 | MAGEL2 | 54551 | RBL1 | 5933 |
| AGT | 183 | FMR1 | 2332 | MAOA | 4128 | REN | 5972 |
| AHO2 | 8121 | FOSB | 2354 | MAPK8IP1 | 9479 | RETN | 56729 |
| AHR | 196 | FOXA2 | 3170 | MC3R | 4159 | RPS6KA3 | 6197 |
| ASRGL1 | 80150 | FOXC2 | 2303 | MC4R | 4160 | RPS6KB1 | 6198 |
| AKT1 | 207 | FOXO1A | 2308 | MECP2 | 4204 | RXRA | 6256 |
| AKT2 | 208 | FRAP1 | 2475 | MEHMO | 8422 | RXRB | 6257 |
| ALMS1 | 7840 | FSHR | 2492 | MEN1 | 4221 | RXRG | 6258 |
| AMACR | 23600 | GABRG3 | 2567 | MIF | 4282 | ACSM3 | 6296 |
| ANCR | 282 | GAD2 | 2572 | MKKS | 8195 | SCARB1 | 949 |
| ANGPTL4 | 51129 | GAL | 51083 | MKRN3 | 7681 | SCD | 6319 |
| ANMA | 80871 | GAMT | 2593 | MMP19 | 4327 | SDC1 | 6382 |
| APOA1 | 335 | GATA2 | 2624 | MRXS11 | 50644 | SDC3 | 9672 |
| APOA2 | 336 | GATA3 | 2625 | MRXS7 | 4429 | SERPINE1 | 5054 |
| APOA4 | 337 | GCCR | 2908 | MT1A | 4489 | SFRP1 | 6422 |
| APOB | 338 | GCG | 2641 | MYC | 4609 | SGBS2 | 23402 |
| APOC1 | 341 | NR3C1 | 2642 | NCB5OR | 51167 | PTPN11 | 5781 |
| APOC3 | 345 | GCK | 2645 | NDN | 4692 | SIM1 | 6492 |
| APOD | 347 | GDF3 | 9573 | NHLH2 | 4808 | SLC2A2 | 6514 |
| APOE | 348 | GDF8 | 2660 | NMU | 10874 | SLC2A4 | 6517 |
| AQP7 | 364 | GFRA2 | 2675 | NPPA | 4878 | SLC6A2 | 6530 |
| AR | 367 | GH1 | 2688 | NPPB | 4879 | SLC6A3 | 6531 |
| ARID5B | 84159 | GHR | 2690 | NPR1 | 4881 | SLC6A14 | 11254 |
| ARNT | 405 | GHRH | 2691 | NPR3 | 4883 | SLC27A1 | 376497 |
| ASPA | 443 | GHRHR | 2692 | NPY | 4852 | SLC27A4 | 10999 |
| ATRN | 8455 | GHRL | 51738 | NPY1R | 4886 | SNRPN | 6638 |
| ATP1A2 | 477 | GHSR | 2693 | NPY2R | 4887 | SOCS3 | 9021 |
| BATF | 10538 | GIPR | 2696 | NPY5R | 4889 | SORBS1 | 10580 |
| BBS1 | 582 | GCG | 2641 | NR5A1 | 2516 | SPARC | 6678 |
| BBS1 | 582 | GNAI1 | 2770 | NR3C1 | 2908 | SREBF1 | 6720 |
| BBS2 | 583 | GNAS | 2778 | NR2F1 | 7025 | STAT1 | 6772 |
| BBS3 | 84100 | GNB3 | 2784 | NRF1 | 4899 | STAT3 | 6774 |
| BBS4 | 585 | GNG3 | 2785 | NRF2 | 4780 | STAT5A | 6776 |
| BBS5 | 129880 | GNRH1 | 2796 | NRLI3 | 9970 | STAT5B | 6777 |
| BBS7 | 55212 | GPAM | 57678 | NR0B2 | 8431 | STK25 | 10494 |
| TTC8 | 123016 | GPC3 | 2719 | NTS | 4922 | TBX3 | 6926 |
| BDNF | 627 | GPC4 | 2239 | NTSR1 | 4923 | TCF4 | 6925 |
| BRS3 | 680 | GPD1 | 2819 | OXT | 5020 | TFAP2A | 7020 |
| BSCL2 | 26580 | GPD2 | 2820 | OXTR | 5021 | TGFB1 | 7040 |
| C3 | 718 | GPR24 | 2847 | SERPINE1 | 5054 | THRA | 7067 |
| CALCA | 796 | GPR7 | 2831 | PARP1 | 142 | THRB | 7068 |
| CLPG | 79104 | GPX1 | 2876 | PAX6 | 5080 | TKT | 7086 |
| CAPN10 | 11132 | GSK3B | 2932 | PCK1 | 5105 | TNF | 7124 |
| CART | 9607 | GTRGEO22 | 91978 | PCSK1 | 5122 | TNFRSF1B | 7133 |
| CAV1 | 857 | H6PD | 9563 | PCSK1N | 27344 | TRH | 7200 |
| RUNX1T1 | 862 | HCRT | 3060 | PDE3B | 5140 | TUB | 7275 |
| CCK | 885 | HCRTR1 | 3061 | PHF6 | 84295 | UBL5 | 59286 |
| CCKAR | 886 | HCRTR2 | 3062 | PIK3R1 | 5295 | UCP1 | 7350 |
| CD36 | 948 | HD | 3067 | PLA2G1B | 5319 | UCP2 | 7351 |
| CDH2 | 1000 | HDH | 25939 | PIP5K2B | 8396 | UCP3 | 7352 |
| CDKN1A | 1026 | HMGA2 | 8091 | PLIN | 5346 | UNC5C | 8633 |
| CDKN1B | 1027 | HRH1 | 3269 | PLSCR1 | 5359 | PDYN | 5173 |
| CEBPA | 1050 | HRH3 | 11255 | PLSCR3 | 57048 | VDR | 7421 |
| CEBPB | 1051 | HSD11B1 | 3290 | PMCH | 5367 | VGF | 7425 |
| CEBPD | 1052 | HSD3B1 | 3283 | PMM2 | 5373 | VLDLR | 7436 |
| CHM | 1121 | HSPA1B | 3304 | PNMT | 5409 | VSX1 | 30813 |
| CHRM3 | 1131 | HTR1B | 3351 | POMC | 5443 | VSX1 | 30813 |
| CIDEA | 1149 | HTR2A | 3356 | PON2 | 5445 | WNT1 | 7471 |
| CLOCK | 9575 | HTR2C | 3358 | PPARA | 5465 | WNT5B | 81029 |
| CNR1 | 1268 | IAPP | 3375 | PPARD | 5467 | WNT10B | 7480 |
| CNTF | 1270 | ICAM1 | 3383 | PPARG | 5468 | WT1 | 7490 |
| VPS13B | 157680 | IDE | 3416 | PPARGC1A | 10891 | WTS | 7492 |
| COL6A2 | 1292 | IDH1 | 3417 | PPARGC1B | 133522 | ZFP36 | 7538 |
| COL8A2 | 1296 | ID2 | 3398 | PRLH | 51052 | MKRN3 | 7681 |
| CORT | 1325 | ID3 | 3399 | PPP1R3A | 5506 |  |  |
| CPE | 1363 | IGF1 | 3479 | PPP1R3C | 5507 |  |  |
| CREB1 | 1385 | IGF2 | 3481 | PPY | 5539 |  |  |

## The adjustment of parameter $\boldsymbol{\alpha}$ in quasi-local similarity indices

We take parameter $\alpha$ as 0.1, 0.01, 0.001, 0.0001, respectively and compare the corresponding precisions. From Fig. S1, the precisions are near maximum when $\alpha$ is 0.001 for hsaPPI and the precisions all get the maximum when $\alpha$ is 0.0001 for the three similarity indices for Corum, HumanNet, String and FunCoup. Thus we take $\alpha$ as 0.001 for hsaPPI and 0.0001 for Corum, HumanNet, String and FunCoup. For the largest network FLN, we take the same value of$\alpha$ as that of the four large networks, *i.e.*, we also take$\alpha$ as 0.0001.


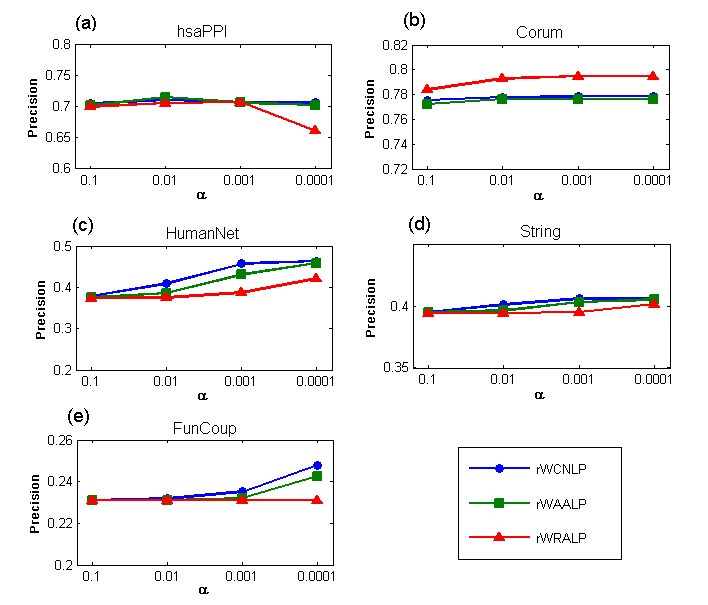


Fig. S1 The adjustment of parameter α in quasi-local similarity indices by link prediction accuracy measured by precision
